# Linezolid-Associated Non-convulsive Status Epilepticus: A Case Report

**DOI:** 10.7759/cureus.104724

**Published:** 2026-03-05

**Authors:** Jonathon Chon Teng Chio, Nimmi Wickramasuriya, Tse Chiang Chen, Jaishree Narayanan, Kareem Elzamly

**Affiliations:** 1 Department of Pediatric Neurology, University of Oklahoma Health Sciences Center, Oklahoma City, USA; 2 Department of Neurology, Tulane University School of Medicine, New Orleans, USA; 3 Department of Neurology, Northwestern University Feinberg School of Medicine, Chicago, USA; 4 Department of Neurology, University of Texas at Houston, Houston, USA

**Keywords:** antibiotic, antibiotic-induced seizures, linezolid, non-convulsive status epilepticus, seizures

## Abstract

Neural, metabolic, and myelosuppressive side effects can occur upon administration of linezolid (LNZ), which is an antibiotic that can cause seizures through multiple mechanisms. Here, we describe the case of a 77-year-old woman who initially presented with a subdural hematoma (SDH). She subsequently developed non-convulsive status epilepticus (NCSE), a category of seizure that can occur secondary to antibiotic use. These episodes persisted despite being treated by multiple anti-epileptic medications, but resolved upon cessation of linezolid. This case is important because it highlights how the appropriate use of a drug can still cause a serious neurological side effect that can negatively impact patient outcomes.

## Introduction

Linezolid (LNZ) is a synthetic oxazolidinone antimicrobial drug indicated for antimicrobial-resistant gram-positive infections [1]. It mediates bacteriostatic effects by binding to bacterial 23S ribosomal RNA of the 50S subunit, which prevents the formation of a functional 70S initiation complex and subsequently hinders the translation of bacterial toxins [2]. The most common adverse effects of LNZ include myelosuppression (with thrombocytopenia (<100,000 platelets/µL) (reference range: 150 to 450 x 103/µL) being the most common), elevated pancreatic enzymes, deranged liver function tests, lactic acidosis, and general symptoms such as headache, nausea, and diarrhea [3,4]. A small number of case reports have reported linezolid-inducing seizures [5-8]. While the mechanism of action in causing seizures is not established, it might involve increased serotonin levels, direct neurotoxic effects, and reduced mitochondrial protein synthesis [9-11]. These pathways have explained other neurological side effects (serotonin syndrome, and peripheral and optic neuropathy) secondary to LNZ.

LNZ is an antibiotic that has been shown to induce seizures, including focal impaired conscious seizures [5-8]. Per the International League Against Epilepsy (ILAE) classification, clinical features of these seizures are sudden behavioral arrest, unresponsiveness, staring, automatisms, being unable to respond appropriately or recall events during the seizure, post-ictal confusion, and potentially with auras that precede the event [12]. Patients with and without seizure history experience seizures within 1-36 days after starting LNZ [5-8]. Multiple pharmacologic approaches were used to treat these seizures with varying degrees of efficacy. The definitive treatment was discontinuation of LNZ, as patients returned to baseline shortly afterward. Notably, a case report by Balkan et al. indicated that LNZ cessation without anti-epileptic treatment was sufficient in preventing seizure recurrence [8]. Here, we describe a case of LNZ-associated non-convulsive status epilepticus (NCSE).

## Case presentation

A 77-year-old woman presented to the emergency department due to a ground-level fall in November 2024 with head trauma. Her past medical history includes heart failure, paroxysmal atrial fibrillation, mitral valve prolapse, sick sinus syndrome, paroxysmal atrial fibrillation, hypothyroidism, adrenal insufficiency, and chronic bilateral lower extremity lymphedema with recurrent cellulitis. The patient is allergic to vancomycin. The patient was also treated with apixaban (Eliquis) in December 2023, but was not taking additional medication that could lower seizure threshold. On admission, the patient's temperature was 87.2°F (30.7°C), blood pressure was 122/54 mmHg, pulse was 68 beats per minute, respiratory rate was 16 breaths per minute, and peripheral oxygen saturation was 99%. She had a Glasgow Coma Scale score of 14 (eyes: 4, motor: 6, verbal: 4). The neurological examination was unremarkable, except for dysarthria. Motor strength was 4/5 throughout, without focal deficits. Due to the patient's low core body temperature, she was given a bear hugger and warming blankets. Initial laboratory tests (basic metabolic panel, urine analysis, and cultures) were notable for blood urea nitrogen (BUN) of 47 milligram/deciliter (mg/dL) (reference range: 6-20 mg/dL) and white blood cell count of 3.8 x 10^9^/L (reference range: 4-11 x 10^9^/L). Glucose and lactic acid were 126 millimole/liter (mmol/L) and 2.4 mmol/L (reference range: <2 mmol/L), respectively. C-reactive peptide was 0.9 milligram/liter (mg/L) (reference range: <1 mg/L). Creatinine (Cr) was 0.73 mg/dL (reference range: 0.5-1.1 mg/dL).

**Figure 1:**
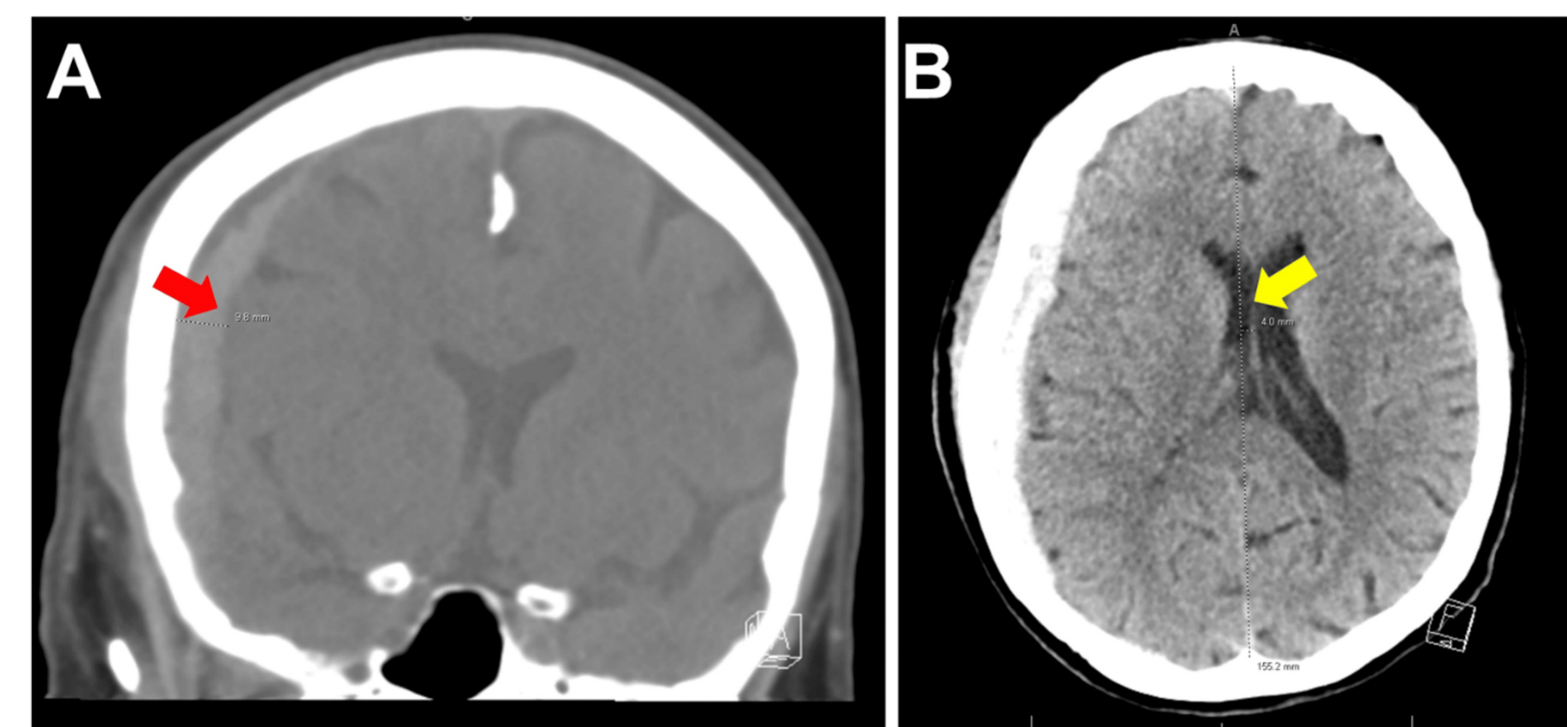
(A) Patient's computed tomography scan of the brain in coronal view demonstrates subdural hemorrhage without fracture or contusion of the skull (red arrow). (B) Axial view of computed tomography scan of the brain provides evidence of a 4-millimeter midline shift (yellow arrow).

Computed tomography (CT) of the head without contrast demonstrated traumatic right hemispheric subdural hemorrhage without skull fracture or contusion and midline shift (Figure 1). Apixaban was discontinued, and neurosurgery did not recommend apixaban loading.

Before her presentation, she had signs of bilateral lower extremities cellulitis in the setting of chronic lymphedema. Ultrasound demonstrated a non-occlusive thrombus on the left internal jugular vein and fluid collection in the right upper extremity. It did not show deep vein thrombosis in the bilateral lower extremities. Therefore, on Day 1, the patient was started on LNZ in 5% (600 mg intravenous every 12 hours) and piperacillin-tazobactam (4.5 g intravenous every eight hours). Infectious disease was consulted, and LNZ was selected due to the patient's allergy to vancomycin.

**Figure 2:**
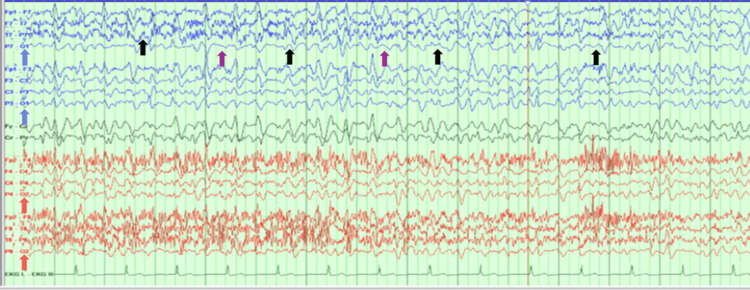
EEG demonstrating non-convulsive status epilepticus. The entire EEG shows slowing of cortical activity, which is indicated by the absence of posterior dominant alpha rhythm in O1 and O2 channels, highlighted by the blue and red arrows, respectively. Furthermore, the black arrows indicate triphasic sharp waves, with the waves in between the black arrows suggesting a slow background (shown with purple arrows). The frequency of the epileptiform discharges was 2.5-3 Hz. EEG: electroencephalography, Hz: Hertz

On the second day, the patient demonstrated worsening neurological function. She was only oriented to herself. While her pupils remained equal and reactive to light, she did not open her eyes to voice and did not follow commands. There was no mydriasis and myoclonus. A repeat CT of the head showed a stable subdural hematoma (SDH) without new acute findings. The patient continued to be confused without improvement in her mental state or neurological examination findings. Another repeat head CT did not show new findings. Therefore, her continued decline in neurological state prompted a continuous electroencephalogram (EEG), which demonstrated frequent bursts of bifrontally predominant rhythmic epileptiform discharges at a frequency greater than 2.5 Hertz lasting approximately two minutes at a time, consistent with NCSE (Figure 2). During interictal periods, the EEG background consisted of theta-range activity. It was disorganized bilaterally without a well-formed posterior dominant rhythm on either side associated with intermittent runs of triphasic waves.

**Figure 3:**
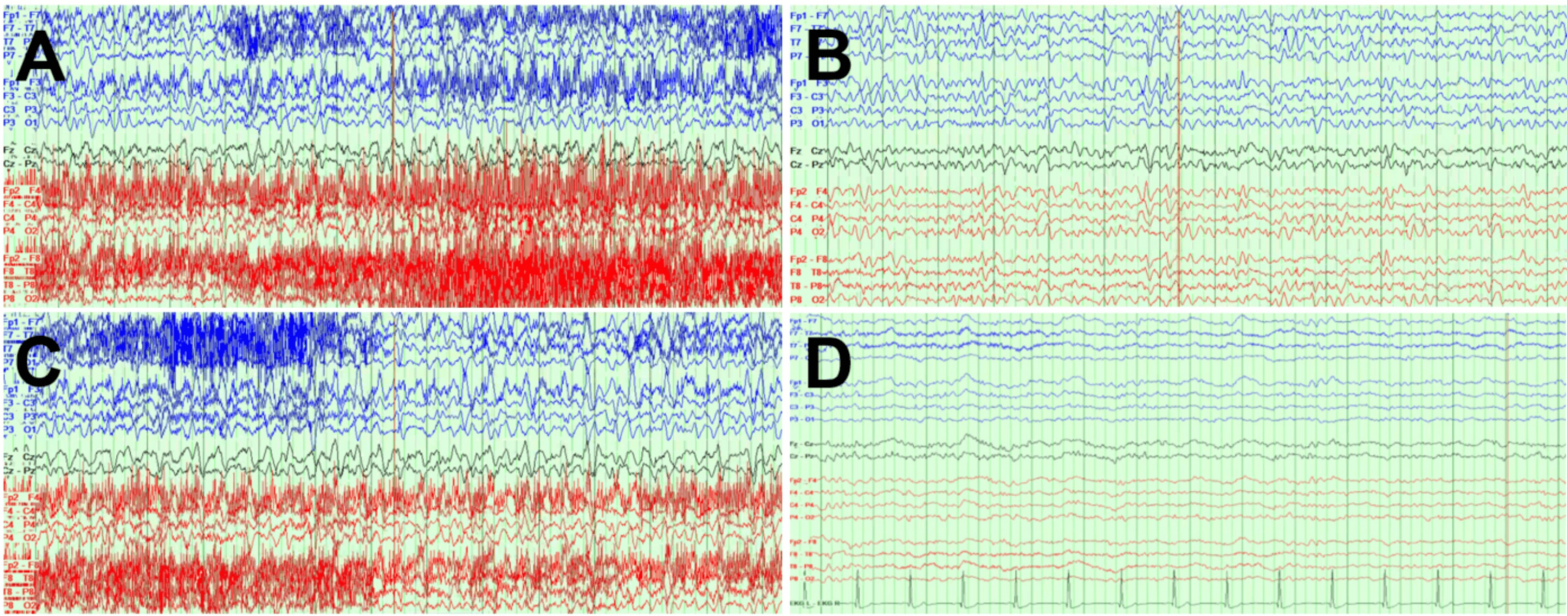
(A) Electroencephalography demonstrates linezolid-associated non-convulsive status epilepticus prior to lorazepam administration. (B) Electroencephalographic findings after lorazepam administration. Clearance of non-convulsive status is a criterion for non-convulsive status epilepticus. However, non-convulsive status epilepticus recurred. There are also linezolid-associated triphasic waves, which are waves with three phases, that do not occur faster than 2.5 Hertz in frequency and hence are not consistent with non-convulsive status epilepticus. Furthermore, the waves do not exhibit anteroposterior lag, as the waves start at the same time in all channels. Waves that exhibit anteroposterior lag are often observed in metabolic etiologies, such as hepatic and renal diseases. (C) The non-convulsive status epilepticus resolved temporarily after lorazepam, but recurred as in A. (D) Linezolid-associated triphasic waves and non-convulsive status epilepticus resolved after linezolid cessation. There is slowing with overlaying fast activity and encephalopathy.

The patient was initially treated with benzodiazepines; however, NCSE quickly returned (Figure 3A, 3C). There were also consistent triphasic waves (Figure 3B).

**Figure 4:**
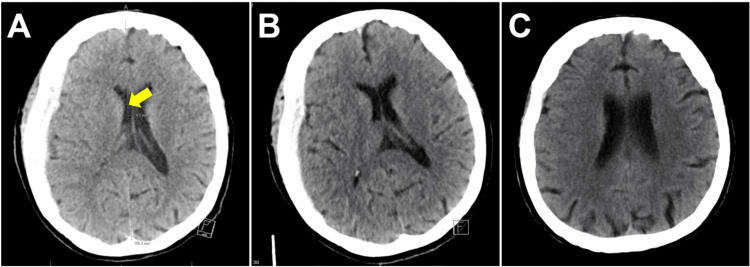
(A) Axial view of computed tomography scan of the brain provides evidence of a 4-millimeter midline shift (yellow arrow). In (B) and (C), repeat scans show interval improvement and resolution, respectively, without the need for neurosurgical intervention.

Treatment was then switched to valproic acid, which was also ineffective. Management was escalated, where lacosamide, levetiracetam, and clonazepam were added. Initially, it was thought that the SDH triggered the seizures, as acute SDH is an established risk factor for seizures and epilepsy [13]. However, since the seizures did not resolve after conventional treatment with antiseizure medications, alternative causes, such as the patient's medication regimen, were considered. Since antibiotic-associated seizures are known, it was hypothesized that the bifrontal rhythmic epileptiform discharges were associated with antibiotics, and the decision was made to discontinue LNZ. Subsequently, her neurological examination improved the following day after stopping LNZ. The patient opened her eyes to voice and became oriented to place and self. The patient's EEG did not show NCSE or triphasic waves (Figure 3D). Moreover, the patient's neurological state continued to improve despite rapid weaning of other antiseizure medications. Upon completely stopping these medications, the patient transitioned toward receiving only levetiracetam. Notably, the patient's platelet count decreased from 143 x 10^3^/µL (reference range: 150-450 x 10^3^/µL) on admission to 67 x 10^3/^µL (reference range: 150-450 x 10^3^/µL) after LNZ initiation but gradually increased to 184 x 10^3^/µL after its cessation. BUN and Cr were 22 and 0.35 mg/dL, respectively. The patient did not experience lactic acidosis (2.6 mmol/L; initial: 2.4 mmol/L) and was not monitored for optic neuropathy. Furthermore, as seen in Figure 4, the patient's initial subdural hemorrhage gradually resolved without neurosurgical intervention during her hospital course.

## Discussion

The literature described multiple potential mechanisms of LNZ-induced seizures. In the context of serotonin syndrome (SS), this is attributed to the drug's reversible and non-selective inhibition of monoamine oxidase (MAO) [9]. Disrupting MAO increases the concentration of epinephrine, norepinephrine, dopamine, and serotonin in the central nervous system (CNS). Elevated serotonin levels can activate multiple serotonin receptor subtypes (particularly 5HT-2A) and lead to SS [14], with seizures as potential clinical manifestations [9].

Increased risk of developing seizures is an adverse effect of antibiotic therapy. Penicillin, cephalosporin, fluoroquinolone, and carbapenem (in particular, imipenem) are drug classes that directly or indirectly antagonize gamma-aminobutyric acid (GABA) receptors, while isoniazid inhibits GABA synthesis [15]. Drug interactions between antiseizure medications and antibiotics have also been shown reduce levels of antiseizure medications and therefore increase seizure risk. For example, carbapenems decrease valproic acid levels by inhibiting the intestinal transporter of valproic acid, preventing efflux of valproic acid from erythrocytes, and increasing valproic acid glucuronidation [16]. Rifampicin reduces levels of lamotrigine, carbamazepine, valproic acid, ethosuximide, and phenytoin by inducing hepatic drug-metabolizing enzymes, in particular, cytochrome P450 and uridine diphosphate glucuronosyltransferase [17]. In addition, CNS injury is an additional risk factor that increases the risk of antibiotic-related symptomatic seizures, as CNS damage can reduce seizure threshold by causing changes in metabolism and neurotransmitter pathways [13]. When suspecting antibiotic-induced seizures, cessation of the offending agent is of utmost importance [18].

**Figure 5:**
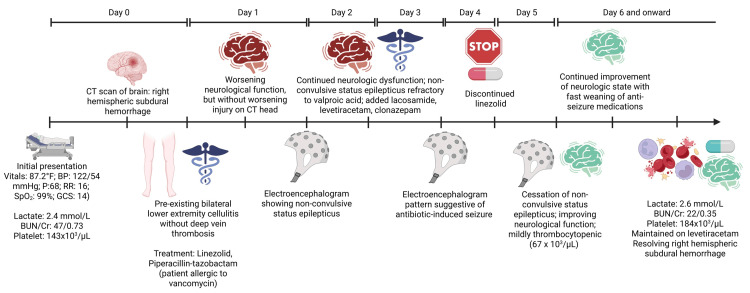
Chronological sequence of key events in the patient's hospital course. This includes antibiotic administration, onset of neurological symptoms, electroencephalogram findings, imaging follow-up, treatment changes, and recovery. BP: blood pressure, P: pulse, RR: respiratory rate, SpO_2_: peripheral oxygen saturation, GCS: Glasgow Coma Scale, BUN: blood urea nitrogen, Cr: creatinine, CT: computed tomography

For our case report, we hypothesized that NCSE was associated with LNZ. Figure 5 illustrates the chronological sequence of events for better correlating drug exposure, EEG changes, and clinical improvement.

The patient has multiple risk and predisposing factors, in particular, SDH, that likely reduced the seizure threshold. Notably, the patient had no previous history of seizures. Therefore, there are structural, metabolic, and inflammatory factors that made the patient particularly susceptible to the neurotoxic effects of LNZ, even when given at the recommended dose. While changes in the patient's mental status can be related to other systemic conditions, such as infections, hospitalization, and frailty, the EEG demonstrated features of NCSE. Notably, piperacillin-tazobactam is also associated with seizures [19]. However, NCSE resolved quickly, and the neurological examination improved upon cessation of LNZ and did not recur despite being weaned off valproic acid, lacosamide, and clonazepam within a relatively short period of time of 48 hours. Piperacillin-tazobactam was discontinued after cessation of LNZ, when the patient did not exhibit symptoms of NCSE. The patient's EEG findings of triphasic waves, frontal rhythmic epileptiform discharges, and generalized slowing align with the electrographic pattern of antibiotic-associated seizures [20]. Due to the patient's overall risk factors that increase seizure likelihood, the patient was maintained on levetiracetam per recommendation from neurosurgery. Overall, the patient's clinical presentation, electrographic findings, and outcomes align with linezolid as a causal possibility and contributing factor to her seizures.

Table 1 provides a comparison of reported cases of linezolid-associated non-convulsive status epilepticus.

**Table 1:** Comparison of reported linezolid-associated non-convulsive status epilepticus cases, including latency, semiology, EEG, management, and outcomes. EEG: electroencephalogram, IV: intravenous, kg: kilogram, LNZ: linezolid, mg: milligram, PO: per oral

Semiology	How was LNZ used?	Latency between seizure development and LNZ treatment	EEG pattern	Seizure management	Reference
A 45-year-old woman with epilepsy on zonisamide, clonazepam, acetazolamide, aripiprazole, and lorazepam developed a postoperative wound infection after excision of a wart-like lesion on the right knee	One dose of IV LNZ, then PO LNZ, dosages unknown	Day 2 of LNZ treatment	Complex partial status epilepticus	LNZ discontinued and IV vancomycin initiated; the patient stabilized with propofol, IV levetiracetam, and home antiepileptic medications; seizures recurred after LNZ was restarted and remained refractory despite benzodiazepines and increased levetiracetam, resolving only after LNZ discontinuation	Shneker et al. (2009) [5]
A 51-year-old man with stage II lung adenocarcinoma treated with cisplatin-vinorelbine and atezolizumab, without prior seizure history, developed a urinary tract infection due to *Enterococcus faecium*	PO LNZ 600 mg	Day 1 of LNZ treatment	Not performed on the day of admission, but the EEG one day later showed absence of epileptic discharge	IV lacosamide 200 mg loading dose, then 100 mg twice daily; replacement of LNZ with vancomycin (unknown dose and method of administration)	Rival et al. (2020) [6]
A 75-year-old man with Alzheimer's disease and epilepsy on levetiracetam developed seven days of painful right elbow swelling associated with a methicillin-resistant *Staphylococcus aureus*-infected stress ulcer	IV LNZ 600 mg every 12 hours, ciprofloxacin 400 mg every 12 hours	Day 5 of LNZ treatment	Complex partial seizure	IV valproic acid (1,200 mg over 30 min) followed by continuous infusion and levetiracetam increased to 1,500 mg/day; ciprofloxacin was discontinued, resulting in transient seizure cessation with relapse 18 hours later and partial response to benzodiazepines; complete seizure resolution after replacing LNZ with IV teicoplanin (400 mg/day)	Cholongitas et al. (2009) [7]
A 68-year-old with methicillin-resistant *Staphylococcus aureus* spondylodiscitis at thoracic level 10-11 with vertebral depression	IV LNZ 100 mg q12 hours	Day 36 of LNZ treatment	Complex partial seizure	LNZ was discontinued, and daptomycin (6 mg/kg/day) was initiated; no antiepileptic therapy was started, and seizures did not recur	Balkan et al. (2015) [8]

## Conclusions

This case underscores the need to recognize antibiotics as having an established, although frequently underrecognized, association with seizures, particularly in patients with underlying CNS pathology. Our patient's course provided compelling evidence that LNZ was associated with NCSE and symptomatic seizures. The episodes persisted despite initially administering lorazepam, escalating antiseizure medications, and ceased after discontinuing LNZ (despite being maintained on piperacillin-tazobactam), which reflects LNZ's neurotoxic potential by inhibiting mitochondrial protein synthesis, increasing serotonergic toxicity, and disrupting GABA pathways. Together, these findings and mechanisms provide a plausible pathophysiological basis for the observed NCSE.

Management of complex drug-associated seizures requires a multidisciplinary team. The careful evaluation of medication profiles, along with close monitoring of therapeutic drug levels and awareness of risk factors, such as preexisting brain injury, is essential. For antibiotics with known neurotoxic potential, monitoring strategies should extend beyond routine laboratory tests to include close surveillance for new or worsening neurological symptoms. For LNZ, clinicians should have a low threshold to evaluate patients presenting with confusion, encephalopathy, or altered mental status, and an EEG may be warranted to detect NCSE. Prompt intervention, including the discontinuation of the offending agent, can significantly improve patient outcomes. Overall, we add to the growing body of literature that associates LNZ with NCSE and emphasize the broader principle that antibiotic neurotoxicity should remain a key differential diagnosis when managing unexplained or treatment-resistant seizures.
